# Extent and Dynamics of Polymorphism in the Malaria Vaccine Candidate *Plasmodium falciparum* Reticulocyte–Binding Protein Homologue-5 in Kalifabougou, Mali

**DOI:** 10.4269/ajtmh.17-0737

**Published:** 2018-05-29

**Authors:** Amed Ouattara, Tuan M. Tran, Safiatou Doumbo, Matthew Adams, Sonia Agrawal, Amadou Niangaly, Sara Nelson-Owens, Didier Doumtabé, Youssouf Tolo, Aissata Ongoiba, Shannon Takala-Harrison, Boubacar Traoré, Joana C. Silva, Peter D. Crompton, Ogobara K. Doumbo, Christopher V. Plowe

**Affiliations:** 1 Malaria Research and Training Center, University of Sciences, Techniques and Technology, Bamako, Mali;; 2 Division of Malaria Research, Institute for Global Health, University of Maryland School of Medicine, Baltimore, Maryland;; 3 Laboratory of Immunogenetics, National Institute of Allergy and Infectious Diseases, National Institutes of Health, Rockville, Maryland;; 4 Division of Infectious Diseases, Department of Medicine, Indiana University School of Medicine, Indianapolis, Indiana;; 5 School of Medicine, Meharry Medical College, Nashville, Tennessee;; 6 Institute for Genome Sciences, University of Maryland Baltimore, Baltimore, Maryland;; 7 Duke University, Durham North Carolina

## Abstract

Reticulocyte-binding homologues (RH) are a ligand family that mediates merozoite invasion of erythrocytes in *Plasmodium falciparum*. Among the five members of this family identified so far, only *P. falciparum* reticulocyte–binding homologue-5 (PfRH5) has been found to be essential for parasite survival across strains that differ in virulence and route of host-cell invasion. Based on its essential role in invasion and early evidence of sequence conservation, PfRH5 has been prioritized for development as a vaccine candidate. However, little is known about the extent of genetic variability of RH5 in the field and the potential impact of such diversity on clinical outcomes or on vaccine evasion. Samples collected during a prospective cohort study of malaria incidence conducted in Kalifabougou, in southwestern Mali, were used to estimate genetic diversity, measure haplotype prevalence, and assess the within-host dynamics of PfRH5 variants over time and in relation to clinical malaria. A total of 10 nonsynonymous polymorphic sites were identified in the *Pfrh5* gene, resulting in 13 haplotypes encoding unique protein variants. Four of these variants have not been previously observed. *Plasmodium falciparum* reticulocyte–binding homologue-5 had low amino acid haplotype (*h* = 0.58) and nucleotide (π = 0.00061) diversity. By contrast to other leading blood-stage malaria vaccine candidate antigens, amino acid differences were not associated with changes in the risk of febrile malaria in consecutive infections. Conserved B- and T-cell epitopes were identified. These results support the prioritization of PfRH5 for possible inclusion in a broadly cross-protective vaccine.

## INTRODUCTION

The efficacy of vaccines targeting *Plasmodium falciparum*, the deadliest human malaria parasite, has been limited in part by genetic diversity in the vaccine antigens in endemic areas,^1,2^ resulting in higher protection against parasites carrying alleles identical to the vaccine genotype.^3–6^ Molecular epidemiological surveys of *P. falciparum* genetic diversity at vaccine-testing sites can identify the most prevalent genetic variants of candidate antigens for consideration in designing broadly protective vaccines and assess the role of specific polymorphisms in strain-specific immunity.^4^

*Plasmodium falciparum* reticulocyte–binding protein homologue-5 (PfRH5) is a promising new blood-stage vaccine antigen that is moving quickly toward clinical development.^7–9^ The invasion of merozoites into the erythrocyte is a multistep process that starts with merozoite binding and reorientation onto the red blood cell (RBC), followed by the formation of a tight junction and culminates with parasite entry into the erythrocyte. Essential to this process is PfRH5, a 63-kDa protein encoded by a gene located in the subtelomeric region of chromosome 4. The gene (PF3D7_0424100) is 1,581 nucleotides long and is refractory to knockout in diverse strains.^10^ The erythrocyte receptor for RH5 is basigin (CD147), and antibodies specific to either RH5 or basigin inhibit erythrocytic invasion by *P. falciparum* in a dose-dependent manner in in vitro tests.^11^ Furthermore, IgG antibodies induced after PfRH5 immunization have higher potency for controlling RBC invasion by both homologous and heterologous *P. falciparum* strains in growth inhibition assays, as compared with IgG raised against the apical membrane antigen-1 (AMA1) and merozoite protein-1, two other leading blood-stage vaccine antigens.^12^

Importantly, the *pfrh5* gene exhibits limited polymorphism across multiple *P. falciparum* laboratory strains^10,13^ and geographic isolates.^14^ Moreover, allelic exchange experiments have determined that specific PfRh5 residues are critical for host erythrocyte invasion. For example, a nonsynonymous change in codon 204 (I204K) converted a poorly invasive strain into a highly invasive one.^10^ In a separate set of experiments, this invasion-enabling single nucleotide polymorphism (SNP) at position 204 changed the protein structure and greatly modified its binding specificity and infectivity.^15^ Taken together, these findings suggest that the functional requirement for erythrocyte invasion exert strong purifying selection on the locus and might constrain the genetic diversity of *pfrh5*. To evaluate the potential clinical implications of this hypothesis and to generate more evidence on the extent of natural diversity in PfRH5 at a single vaccine-testing field site, we investigated the prevalence and dynamics of *pfrh5* polymorphisms during acute malaria episodes in Malian children and adults. Longitudinal analyses were carried out to assess whether specific *pfrh5* alleles, or changes at this locus in consecutive infections, are associated with the development of malaria symptoms. This prospective study aimed to identify specific PfRH5 amino acid residues that are critical in clinical malaria, and to identify conserved B- and T-cell epitopes that should be taken into account in the design of a malaria vaccine intended to provide broader, strain-transcending protection against diverse parasites than was achieved with first-generation blood-stage malaria vaccines.

## MATERIALS AND METHODS

### Sample origin.

**Figure 1.**
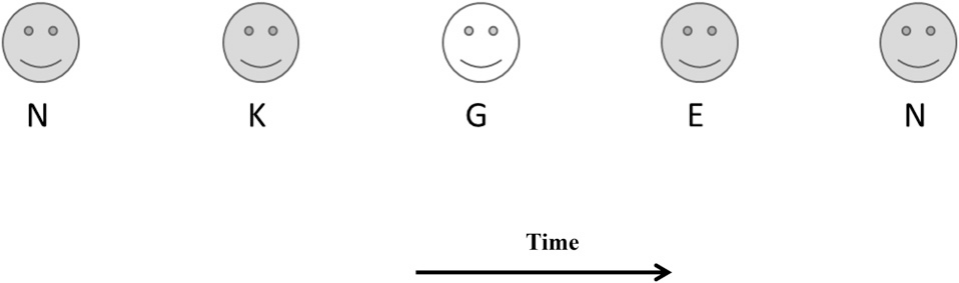
Within-host dynamics of *Plasmodium falciparum* reticulocyte–binding homologue-5 (PfRH5) amino acid polymorphisms during consecutive malaria infections. The gray color represents asymptomatic infections, and the white color represents clinical malaria. Different abbreviations (Asparagine: N, Lysine: K, Glycine: G, and Glutamate: E) indicate different alleles at a polymorphic amino acid site within PfRH5. In this hypothetical example, at this amino acid position, the individual goes from being asymptomatic during his first two infections (N then K) to symptomatic during his third infection (G). The individual is later infected with variants harboring different amino acids than the previous two infections but in one instance similar to the first infection (N).

Samples used in this study originated from Kalifabougou, a rural village located 45 km northwest of Bamako, Mali. Malaria transmission is seasonal (June–December) and hyperendemic, with seasonal peaks following the rains in July through October. The study population and clinical parameters have been described previously.^16^ Briefly, 695 healthy children and adults, aged 3 months to 25 years, were enrolled in this ongoing study beginning in May 2011. Samples and clinical data for this study were collected from May 2011 through January 2012. Study participants were surveyed during scheduled visits occurring every 2 weeks and anytime they presented with malaria-like symptoms. During surveillance, clinical parameters were measured and malaria parasites were detected by thick smears. For this study, a clinical malaria episode was defined as any parasitemia by blood smear with a temperature greater or equal to 37.5°C. Participants with positive blood smears were treated according to the National Malaria Control Program guidelines in Mali. Peripheral whole-blood samples were collected by finger stick in capillary collection tubes (Sarstedt, Newton, NC) for the cryopreservation of the cell pellet and/or as dried blood spots (DBS) on 903 filter paper (Whatman/GE Healthcare, Piscataway, NJ). Longitudinal polymerase chain reaction (PCR) analysis of DBS collected at scheduled visits was performed as previously described.^17^ Whole-blood samples from participants with at least two malaria episodes during the season were selected for genomic DNA (gDNA) extraction as described below (Figure 1).

### DNA extraction and genotyping.

Whole-blood gDNA was extracted from blood samples and DBSs using the QIAmp 96 DNA kit (Qiagen, Valencia, CA). To genotype polymorphisms in *pfrh5*, a nested-PCR was used to amplify the major exon in all the samples. Briefly, primers F1 (5′ GCAATAAAAAAAACGAAGAATCAAGA 3′) and R1 (5′ TGAAAATATTCCATTTTAATTGGGAC 3′) were used in the primary PCR reaction (1,465 bp). Conditions for this amplification were 94°C for 5 minutes, followed by 35 cycles of denaturation at 94°C for 1 minute; primer annealing at 48°C for 1 minute, and extension at 60°C for 1.5 minutes with a final extension at 60°C for 5 minutes. PCR products from the first amplification were subsequently used as a template for a second round of amplification (1,418 bp) with primers F2 (5′ GAAAATAATCTGACGTTACTACCA 3′) and R2 (5′ GACATCATTGAACTTCATTTGTAG 3′). Amplification conditions were the same as in the primary amplification with only 10 cycles instead of 35. PCR reactions were carried out in a total volume of 50 μL with 47 μL of Platinum Super Mix in 96-well plates (Invitrogen, Carlsbad, CA), 1 μL of each primer (1 μM final concentration) and 1 μL of malaria parasite DNA. Secondary PCR had 47 μL of Platinum Mix, 1 μL of each (1 μM final concentration) primer and 1 μL of primary PCR product. Secondary PCR products were electrophoresed on a high-throughput precast gel system (Invitrogen), then visualized and photographed under ultraviolet light with ethidium bromide as the stain.

PCR products were purified using filter plates (Edge Biosystems, Inc., Gaithersburg, MD) attached to a vacuum manifold and eluted in water, and sequenced in a 10 μL reaction using BigDye v3.1 (Applied Biosystems, Carlsbad, CA). The sequencing products were ethanol precipitated and run in 7 μL HiDi formamide on an ABI 3730XL 96-capillary sequencer (Thermo Fisher Scientific, Waltham, MA). Following sequencing, fragments were assembled, edited and aligned to *P. falciparum* 3D7 reference (GenBank accession number: XM_001351508) sequence using Sequencher v.5.0 (Gene Codes Corporation, Ann Arbor, MI). Infections were classified as single/predominant allele infections or as multiple allele infections if the secondary peak height was greater than 50% of the primary peak height at any polymorphic position. Sequence alignment was performed using Bioedit version 7.^18^

### Population genetics analysis.

Haplotypes were defined as an uninterrupted sequence of nucleotides or amino acid sequence variants, and the *pfrh5* locus of the *P. falciparum* 3D7 strain (GenBank accession number: XM_001351508) was used as the reference sequence for all the comparisons. The nucleotide diversity at the locus, among sequences generated from samples collected during the study, was assessed by estimating the parameter π, the average number of nucleotide differences per site between two random sequences.^19^ Bootstrap resampling was used to simulate the distribution of π and to calculate confidence intervals (CIs). These analyses were conducted using MEGA6^20^ and DnaSP v5.^21^ The prevalence of nonsynonymous substitutions was estimated using SAS v9.1 (SAS Institute Inc., Cary, NC). To assess if regions of *Pfrh5* evolve under neutrality (Kimura 1983), the McDonald and Kreitman test was measured using *Plasmodium reichenowi* (PRCDC_0322700) as an outgroup, and analyses were performed using DnaSP v5.^21^ The hypothesis that positive selection has occurred in PfRH5 branch/branches tree was assessed by applying the branch-site test^22^ and the branch-site unrestricted statistical test for episodic diversification (BUSTED) implemented in the HYPHY package.^23^ Although BUSTED identifies the effect of sporadic positive selection on a subset of branches in the lineage, the branch-site test uses a χ^2^ distribution with one degree of feedom to compare a model, which allows positive selection on one or more variants of a group to a model that does not permit positive selection (*P* < 0.05 for sites positively selected). Tajima’s *D* statistic was also calculated, with a positive value of Tajima’s *D* indicating an excess of alleles at intermediate frequency, consistent with balancing selection, and negative values consistent with directional selection or with rapid population expansion following a bottleneck. To assess recombination events in *pfrh5*, which may result in negative Tajima’s *D* values and lead to a false inference of directional selection or rapid expansion, an estimate of recombination (R) and the minimum number of recombination events (RM)^24^ in the data were estimated using DnaSP v5. These recombination events were confirmed by the genetic algorithm recombination detection test.^25^ Genetic association between polymorphic sites and the effect of intragenic recombination on sequence polymorphism were assessed using the Z_ns_^26^ and the ZZ^27^ statistics, respectively.

To group PfRH5 sequences, a sequence clustering analysis was conducted. Structurama, a Bayesian clustering algorithm software,^28^ which treats the number of populations as a random variable, was used to determine the posterior number of populations (K). We identified two populations by this Dirichlet process. We then used Structure version 2.3.3^29^ to assign Rh5 sequences to haplotype groups. Ten runs of 50,000 burn-ins and 100,000 iterations were performed for *K* = 2 using an admixture model.

### Within-host amino acid dynamics.

The frequency of protein sequence variants, or haplotypes (*N* = 13), and of amino acid residue polymorphic sites (*N* = 10) were compared in clinical and asymptomatic malaria cases using a χ^2^ test while correcting for multiple measurements (two side *P* values: 0.05/13 = 0.003 and 0.05/10 = 0.005, respectively). Generalized estimating equations were used to perform logistic regression to estimate whether specific *pfrh5* alleles or shifts in *pfrh5* alleles were associated with the development of clinical symptoms in an individual’s consecutive infections. Both single- and multiple-clone infections were included in the analysis. The primary outcome was whether an individual’s second of two paired consecutive infections was symptomatic or asymptomatic, with the presence of infection being defined based on the results of the *pfrh5* PCR (Figure 1). Study participants’ paired consecutive episodes were classified as follows: absence of infection to asymptomatic (OA, *N* = 40); absence of infection to symptomatic (OS, *N* = 9); asymptomatic to asymptomatic (AA, *N* = 58); asymptomatic to symptomatic (AS, *N* = 16); symptomatic to asymptomatic (SA, *N* = 13); and symptomatic to symptomatic (SS, *N* = 3). The primary predictor for the analysis was the presence of a specific *pfrh5* variants or whether there was a change in amino acid sequence variant in the next consecutive infection. The models included potential confounding variables such as age group, or symptoms in the first of the two infections. Analysis was performed using SAS v9.1.

### B- and T-cell epitopes.

*Plasmodium falciparum* reticulocyte–binding homologue-5 linear B-cell epitopes were predicted using the ABCpred server (http://www.imtech.res.in/raghava/abcpred) using a threshold of 0.85. A T-cell epitope prediction algorithm (NetMHCpan-3.0, http://www.cbs.dtu.dk/services/NetMHCIIpan-3.0) was used to screen PfRH5 sequences for potential CD4^+^/CD8^+^ epitopes with respect to the most frequent human leukocyte antigen (HLA) allele frequencies in Mali.^30,31^ Disordered regions (intrinsically unstructured regions [IURs]; over-representation of few residues with low predicted secondary structure) were identified using the regional order neural network (RONN) server (http://www.strubi.ox.ac.uk/RONN). A putative epitope cutoff was set at 50 nM (high affinity binding).

### Human subject protections.

The study protocols were reviewed and approved by the institutional review boards of the University of Sciences, Techniques, and Technology of Bamako, Mali; the University of Maryland Baltimore; and the National Institute of Allergy and Infectious Diseases. The study participants gave their consent and/or assent to participate in the study.

## RESULTS

### Data summary.

Blood spots on filter paper and/or whole-blood specimens (*N* = 312) were selected from the incidence study sample repository. Samples had been collected biweekly from individuals with at least two malaria episodes during study follow-up starting at the beginning of the 2011 malaria transmission season. Of these specimens, 198 (63.5%) were successfully sequenced using primers specific to the *Pfrh5* gene. The dynamics of consecutive malaria infections and clinical episodes was assessd using 139 sequences generated from paired-consecutives episodes as described in Table 1. Amino acid sequence variants were defined based on single amino acid polymorphisms. Of the 198 sequences generated (GenBank accession number: MG012489–MG012686) and based on the quality of the sequences, all individuals had only one haplotype.

**Table 1:** Dynamics of malaria infections and clinical episodes in male and female consecutive infections according to age groups.

Characteristic	Male	Female	Male/Female ratio
OA			
≤ 5 years	17	16	1.06
> 5 years	2	5	0.40
OS			
≤ 5 years	4	2	2
> 5 years	2	1	2
AA			
≤ 5 years	24	25	0.96
> 5 years	4	5	0.80
AS			
≤ 5 years	6	8	0.75
> 5 years	1	1	1
SA			
≤ 5 years	6	3	2
> 5 years	3	1	3
SS			
≤ 5 years	2	1	2
> 5 years	0	0	0
Total	71	68	1.04

Study participants’ paired consecutive episodes were classified as follows: absence of infection to asymptomatic (OA); absence of infection to symptomatic (OS); asymptomatic to asymptomatic (AA); asymptomatic to symptomatic (AS); symptomatic to asymptomatic (SA); symptomatic to symptomatic (SS).

### Polymorphisms and haplotype prevalence of *pfrh5* gene.

**Figure 2.**
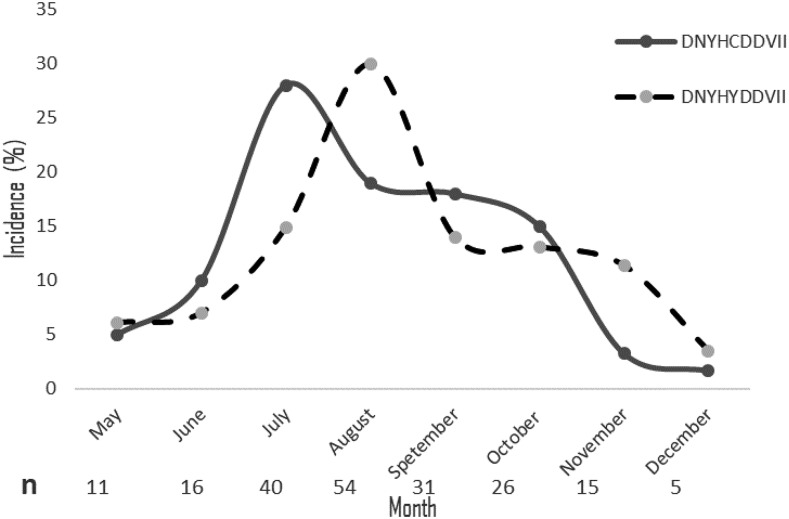
Dynamics of *Plasmodium falciparum* reticulocyte–binding like homologous protein 5 haplotype during 8 months of follow-up. Incidence of the two major RH5 haplotypes from May to December in Kalifabougou, Mali. “*n*” represents the sample size per time point, and bracket are error bars.

Ten nonsynonymous polymorphisms were identified in *pfrh5*. These mutations and the amino acid residues in which they are present are described as follows: D67E, N117S, Y147H, H148D, C203Y, D249Y, D305G, V371I, I407V, and I410M. The most common polymorphisms were C203Y (66.8%) followed by H148D (5%). Based on these 10 amino acids polymorphisms, 13 unique protein variants were identified (Table 2). Seven of these variants were seen only once in the current dataset. The most frequent variant, which we define by the composition at the 10 variable residue positions (variant A: DNYHYDDVII), had a prevalence of 57.3% whereas the frequency of the second most prevalent variant (Variant B: DNYHCDDVII) was 30.7% (Table 2). An analysis by the Dirichlet process using Structurama identified these two groups as the main populations in this transmission site. An analysis of the distribution and dynamics of variants during the 8 months of follow-up showed that the incidence of variants A and B were only different in July (*P* = 0.03, χ^2^ = 4.5). Interestingly, the incidence of the two major haplotypes fluctuate slightly and alternate over time (Figure 2), which is characteristic of frequency-dependent selection. Also, no difference was seen in the incidence of the C203Y and the H148D mutations during follow-up (χ^2^ = 8.01, *P* = 0.33; and χ^2^ = 3.18, *P* = 0.86, respectively).

**Table 2:** Reticulocyte-binding like homologous protein 5 (PfRH5) variant frequency and single nucleotide polymorphisms in Mali isolates

		D67E	N117S	Y147H	H148D	C203Y	D249Y	D305G	V371I	I407V	I410M	
Protein variant	Variant	–	–	–	–	–	–	–	–	–	–	Frequency (%)
DNYHYDDVII	A	D	N	Y	H	C	D	D	V	I	I	57.3
DNYHCDDVII	B	–	–	–	–	Y	–	–	–	–	–	30.7
DNYHYDDVVI	C	–	–	–	–	Y	–	–	–	V	–	3
DNYDYDDVII	D	–	–	–	D	Y	–	–	–	–	–	2.5
DNHDYDDVII	E	–	–	H	D	Y	–	–	–	–	–	2
DNYHCDDVVI	Other	–	–	–	–	–	–	–	–	V	–	1
DNYDYDDVVI	Other	–	–	–	D	Y	–	–	–	V	–	0.5
DNYHCDDVIM	Other	–	–	–	–	–	–	–	–	–	M	0.5
DNYHCYDVII	Other	–	–	–	–	–	Y	–	–	–	–	0.5
DNYHYDDIII	Other	–	–	–	–	Y	–	–	I	–	–	0.5
DNYHYDGVII	Other	–	–	–	–	Y	–	G	–	–	–	0.5
DSYHCDDVII	Other	–	S	–	–	–	–	–	–	–	–	0.5
ENYHYDDIII	Other	E	–	–	–	Y	–	–	I	–	–	0.5
Prevalence of the major amino acid	–	99.5	99.5	98	95	67	99.5	99.5	99	95.5	99.5	–

PfRH5 = *Plasmodium falciparum* reticulocyte–binding homologue-5.

Polymorphic codons of *Pfrh5* gene compared with the 3D7 strain (XM_001351508). Haplotype prevalences are reported in the right panel. The most prevalent amino acid frequency is listed in the bottom panel.

Genetic diversity parameters were estimated for the whole *pfrh5* gene. At this study site, *pfrh5* nucleotide diversity (π ± SD) and haplotype diversity (*h* ± SD) were 0.00061 ± 0.00005 and 0.58 ± 0.027, respectively. All polymorphisms in the data occurred at nonsynonymous sites, with π_S_ = 0 and π_N_ = 0.00075. The average number of pairwise nucleotide differences between sequences in this sample set was 0.74.

To assess whether positive selection might be responsible for the low diversity observed in *pfrh5* and influence its eventual use as a vaccine candidate, the McDonald and Kreitman test was conducted with *P. reichenowi* used as a source of the divergence estimates to be compared with polymorphism in *P. falciparum*. Despite the higher rate of nonsynonymous polymorphism relative to synonymous polymorphism, the test does not support a departure from neutral expectation (two-tailed *P* value = 0.55). This may result from the lack of power due to the small number of polymorphic sites, which was exacerbated by the elimination from the test of three codons with multiple variable positions. Moreover, the estimate of the coefficient of variation for α_s_ generated using a maximum likelihood model (gamma distribution with shape 1) was small (< 0.000001) inferring a constant evolution rate among sites. Finally, the Tajima’s *D* statistic did not show clear departure from neutrality (Tajima’s *D* = −1.32; as a rule of thumb, a *D* statistic with absolute value less than 2 is not considered significant). Despite not being significant, the strong negative Tajima’s *D* value may be suggestive of a rapid population expansion following a bottleneck.

Recombination is a process that creates new haplotypes by reassorting variants of a locus. These novel haplotypes may be different from the vaccine allele(s), limiting its efficacy. To evaluate this second source of genetic diversity in the dataset, the contribution of recombination to *pfrh5* diversity was estimated. Sequence analyses suggested that the overall recombination parameter (R), which is the recombination rate per generation between the most distant sites of *Pfrh5*, was 77.3. An estimate of R between adjacent sites of *Pfrh5* was 0.064, whereas the minimum number of recombination events (Rm) in the history of the dataset was one. These findings are indicative of meiotic recombination occurring within the *Pfrh5* locus. Values of the genetic association estimated between polymorphic sites (Zns) and the effect of intragenic recombination (ZZ) on sequence polymorphism were 0.0216 and 0.0256, respectively, indicative of low linkage disequilibrium between polymorphic sites.

### Dynamics of amino acids in relation to malaria symptoms.

Participants who had clinical malaria experienced one to five malaria episodes during the 9-month study period. Nearly half (48%) of these participants experienced three malaria episodes. A logistic regression model was used to estimate whether specific *pfrh5* alleles were associated with the development of clinical symptoms in consecutive infections. The primary predictor for the analysis was the presence of a specific *pfrh5* allele. Only amino acid 203 had enough changes during follow-up to assess the dynamics of codons in relation to the occurrence of malaria clinical episode. Results from the regression models for amino acid 203 (95% CI: −1 to 1.1) analysis did not show an association between amino acid residue at position 203 and the occurrence of clinical malaria episode in consecutive *P. falciparum* infections. Finally, a comparison of mean parasite density in mutated (203Y: 20852; 95% CI: 14,390–27,313) and wild type (203C: 33745; 95% CI: 19,340–48,150) parasites at position 203 revealed that neither of the amino acids at this site was associated with the level of parasitemia.

### *Pfrh5* variants and malaria clinical episodes.

To investigate whether individual codons or PfRH5 variants affected the risk of clinical malaria episodes, association between the most prevalent PfRH5 variants and residues and the incidence of clinical malaria was evaluated. Neither variant A (χ^2^ = 2.46, *P* = 0.12) nor variant B (χ^2^ = 2.45, *P* = 0.11) was associated with malaria risk. Furthermore, the incidences of two of the most prevalent amino acid polymorphisms, H148D and C203Y, were identical ([χ^2^ = 0.001, *P* = 0.97] and [χ^2^ = 5.30, *P* = 0.02], respectively) in study participants when malaria clinical cases were compared with asymptomatic infections (*P* value corrected for multiple comparisons, *P* = 0.05/10 = 0.005).

### Antigenic diversity of B-cell and T-cell epitopes within PfRH5.

**Figure 3.**
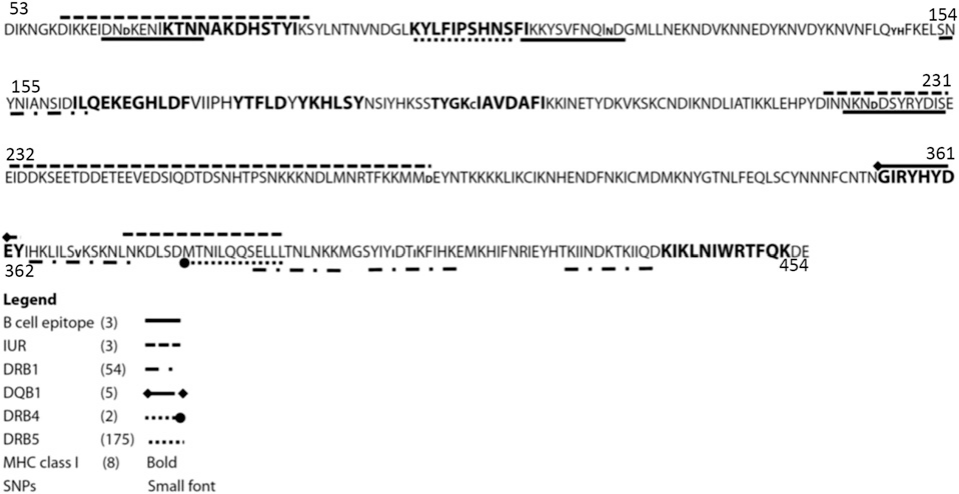
*Plasmodium falciparum* reticulocyte–binding homologue-5 peptide locations within the gene. Polymorphic residues are in lower case font. B-cell epitopes are underlined in plain black whereas HLA Class II (antigen D related beta chain [DRB]1, and DQB1) epitopes are underlined, respectively with (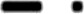) and (
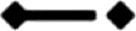
) dashed lines. Dashed (
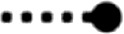
) and (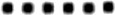) lines are shown under HLA Class II DRB4 and DRB5 epitopes, respectively. MHC class I epitopes are bolded. Number in parenthesis represent the total number of epitopes identified in this dataset. Legends. IUR = intrinsically unstructured regions; SNPs = single nucleotide polymorphism.

To ascertain whether a subset of epitopes may replace the whole PfRH5 antigen as a conserved immunogen, IURs in addition to B- and T-cell epitopes were identified and screened for the presence of polymorphisms. Three B-cell epitopes, eight major histocompatibility complex (MHC) class I-restricted T-cell epitopes, 175 MHC class II-restricted T-cell epitopes, and three IURs (highly disordered) were identified (Figure 3, Table 3). Eight of 10 mutations in the *pfrh5* gene were located in antigenic/epitope regions (χ^2^ = 8.1, *P* = 0.004) (Figure 3), revealing a significant polymorphisms enrichment in regions of immunogenic potential. A conserved region (amino acid residues 250–304) located in the middle of the gene was highly disordered (over-representation of few residues with low predicted secondary structure) but also enriched in B-cell epitopes.

**Table 3:** Potential high binding (IC50 < 50 nM) CD8^+^ T-cell, CD4^+^ T-cell, and B-cell epitopes in *Plasmodium falciparum* reticulocyte–binding homologue-5

Allele	Predicted peptide	IC50 (nM)	MHC restriction	Number of polymorphic sites
T-cell epitopes				
			Class I	
HLA-A*23:01	KYLFIPSHNSFI	9.08		0
HLA-A*30:01	KIKLNIWRTFQK	16.15		0
HLA-B*35:01	YTFLDYYKHLSY	19.23		0
HLA-B*35:01	FVIIPHYTFLDY	22.85		0
HLA-A*30:01	GIRYHYDEYIHK	24.39		0
HLA-A*02:01	ILQEKEGHLDFV	27.43		0
HLA-A*23:01	TYGK**C**IAVDAFI	38.75		1
HLA-A*30:01	KTNNAKDHSTYI	44.27		0
			Class II	
DRB1_1304	KLIL**S**VKSKNLN	33.82		1
	SYIY**I**DT**I**KFIH	35.44		2
	HKLILS**V**KSKNL	36.13		1
	TNILQQSELLLT	40.87		0
	GSYIY**I**DT**I**KFI	43.09		2
	**I**KFIHKEMKHIF	43.28		1
	SELLLTNLNKKM	44.77		0
	TKIINDKTKIIQ	45.48		0
DRB1_0701	KYLFIPSHNSFI	32.95		0
	YLFIPSHNSFIK	39.41		0
	KLILS**V**KSKNLN	40.14		1
	HKLILS**V**KSKNL	44.98		1
	SNYNIANSIDIL	49.01		0
DRB1_1101	YIHKLIL**S**VKSK	29.07		1
	HKLILSVK**S**KNL	30.62		1
	KLIL**S**VKSKNLN	33.20		1
	LKYLFIPSHNSF	35.97		0
	EYIHKLIL**S**VKS	34.24		1
	KYLFIPSHNSFI	41.07		0
	IHKLIL**S**VKSKN	41.24		1
	**I**KFIHKEMKHIF	44.58		1
DRB1_0120	KYLFIPSHNSFI	8.430		0
	KLIL**S**VKSKNLN	8.80		1
	LKYLFIPSHNSF	9.77		0
	HKLILSVKSKNL	11.39		0
	LILSVK**S**KNLNK	11.66		1
	GLKYLFIPSHNS	12.49		0
	YLFIPSHNSFIK	12.23		0
B-cell epitopes				
	KKYSVFNQI**N**DG			1
	DSYRYDISEEID			0
	EIDN**D**KENIKTN			1

## DISCUSSION

*Plasmodium falciparum* reticulocyte–binding homologue-5 is a conserved malaria vaccine candidate that induces antibodies that are protective in in vitro assays,^32^ non-human primate studies^7^ and field studies.^16,33^ *Plasmodium falciparum* reticulocyte–binding homologue-5 interacts with basigin (CD147), an erythrocyte surface protein, to allow the invasion of the malaria parasite into the erythrocyte. The goal of this study was to assess the genetic diversity, haplotype prevalence, and dynamics of the PfRH5-encoding gene to guide the selection of variants and/or B- and T-cell epitopes that are representative of the parasite population of this study site for possible inclusion in a broadly effective malaria vaccine. Examining the dynamics of antigen diversity in prospective cohort studies can show evidence to guide the design of vaccines that will be efficacious against diverse targets. Clinical trials have shown that some malaria vaccines based on a single strain induce protection that is specific to the vaccine strain.^5,34–36^ Likewise, pneumococcal^37^ and influenza^38^ vaccine trials have shown the importance of antigen polymorphisms on vaccine efficacy and the selection of variants to include in vaccine formulations.

In the present study, we analyzed 198 sequences from Kalifabougou, Mali. Power calculations based on sequences extracted from MalariaGEN database show that, with this sample size, we have 83.6% power to identify 85.7% of all alleles present, suggesting that this sample size provides a reliable assessment of population diversity. Two protein variants that represent most of the circulating alleles in this region were identified. In support of previous findings based on small numbers of parasite strains,^10,11,39^ our large field study confirmed that *pfrh5* was highly conserved in a setting where other vaccine antigens show extensive polymorphism. The nucleotide diversity, as measured by π, was very low (0.00061 ± 0.000012) compared with the diversity observed in the locus encoding the well-known blood-stage AMA1 (0.0163) from the same geographical region.^40^ Despite the high rate of polymorphism in nonsynonymous relative to synonymous sites, usually suggestive of positive selection, none of the statistical inference methods used to detect departure from neutrality was indicative of evolution under immune pressure, possibly because the tests were underpowered to detect it in this dataset. In addition, recombination events that may introduce new protein sequence variants in the locus were very low (only one event observed), as indicated by low R, Rm, Zns, and ZZ statistics. The low effective recombination rate may also be explained by the limited number of variable sites that are not singletons, which prevents the detection of recombination events. The very low values of nucleotide diversity not only in nonsynonymous (π_NS_) sites but also in silent sites (π_S_) and observed in this dataset are usually indicative of a very recent common ancestor to all sequences, as one might expect from a hard sweep or a recent, strong bottleneck. This observation is consistent with a previous study that suggests that a small chromosomal region containing *Pfrh5* and three other genes was recently acquired via lateral gene transfer from another *Plasmodium* lineage, possibly an ancestor of *Plasmodium adleri*,^41^ an event that would impact polymorphism similarly to a very recent bottleneck.

Ten nonsynonymous SNPs were identified in *pfrh5*, which gave rise to 13 protein variants with frequencies ranging from 0.5% to 57%. In comparison, using sequences from the blood-stage vaccine candidate AMA1, 186 unique amino acid haplotypes were identified in Bancoumana (prevalences ranging from 0.3% to 2.54%)^40^ and 214 unique haplotypes were observed in Bandiagara (prevalences from 0.2% to 3.6%),^5^ two other towns in Mali. *Plasmodium falciparum* reticulocyte–binding homologue-5 amino acid polymorphism C203Y, located near the boundary of the region involved in the PfRH5–basigin interaction was the most variable residue observed in this dataset. None of the codons within PfRH5-basigin interface (S197, I204, N347, Y358, and E362)^42^ were polymorphic. The conservation of these residues is suggestive of the functionally critical role they play in the interaction between PfRH5 and basigin to allow parasite invasion into RBCs. The overall conservation of *pfrh5* is highly unusual for what would be expected of a gene with high expression during the blood-stage and for which the product is exposed to the host’s immune system, and therefore more inclined to exhibit a high degree of amino acid diversity.^43,44^ However, as mentioned previously, these observations may be more reflective of the unusual evolutionary history of the *pfrh5* locus in *P. falciparum*^41^ than of its interactions with the human immune system.

It is also possible that the protein has limited exposure to the immune system as it has to form a complex with *P. falciparum* reticulocyte–binding protein homologue-5-interacting protein^45^ and P113.^46^ Therefore, polymorphisms in this protein may be found at flexible or exposed sites. Findings from Kenya^47^ and Mali,^16^ suggest that anti-PfRH5 antibody concentrations were very low in malaria-exposed subjects compared with other blood-stage antigens, providing support for the notion that PfRH5 is either non-immunogenic or that its immunogenicity is undermined by mechanisms that reduce immune recognition or response. The low antigenicity of PfRH5 in natural settings will likely require the use of a potent adjuvant in a RH5-based vaccine to induce the high antibody levels needed for protection.

Using naturally-occurring clinical malaria episodes, we assessed whether specific *pfrh5* alleles were associated with clinical disease, suggesting that certain alleles may allow for more efficient erythrocyte invasion and potentially more virulent infections. This study did not identify amino acids that were associated with the parasite’s ability to invade RBCs, at least as measured by association with clinical outcomes. All residues at position 204 in this dataset were shown to be wild type, suggesting a high relative fitness of an isoleucine (I) in this position. Although this *post hoc* analysis was based on a relatively small number of paired consecutive infections, the very low degree of polymorphism in PfRH5 compared with other blood-stage antigens supports the conclusion that not enough time has occurred for genetic variants to emerge.

Identifying epitopes that are conserved and localized in intrinsically unstructured regions of PfRH5 has important implications for vaccine design. Our study revealed 21 B- and T-cell epitopes that are conserved and located in disordered regions across all the sequences from the Kalifabougou site. Based on our data, the inhibition activity of these epitopes and/or fragments should be assessed by functional assays to ascertain their relevance as candidate epitopes of a PfRH5-based vaccine.

We have advocated routinely assessing whether polymorphism in vaccine candidates is immunologically important and therefore relevant to vaccine development. The data reported here on the diversity and dynamics of this antigen’s alleles and their association with the hazard of clinical malaria episodes may inform the design of a PfRH5-based vaccine or, more likely, of a multivalent, multistage malaria vaccine that improves on the modest efficacy of the first-generation vaccines.^48^ The results of this study suggest that polymorphism in PfRH5 is very limited, stable during consecutive infection that occur over the course of a malaria transmission season and not associated with clinical risk. This high degree of conservation and lack of association between genetic variation and clinical disease is in sharp contrast to other leading blood-stage vaccine antigens whose efficacy was constrained by allelic escape.^3,5,34,49,50^ Although encouraging for PfRH5’s prospects as a vaccine, even this limited degree of polymorphism allows for the possibility of vaccine escape and allele-specific efficacy under the intense selection of vaccination as supported by the apparent signal of frequency-dependent selection. Moreover, although changes in the *P. falciparum* circumsporozoite protein (CSP) were not associated with clinical risk in paired consecutive infections,^51^ the CSP-based vaccine RTS,S nevertheless did have allele-specific efficacy.^6^ If the predominant alleles identified in this study are confirmed to predominate in other epidemiological settings, it would be prudent to include both alleles in a vaccine containing PfRH5 to reduce the effect of allele-specific efficacy.

## References

[1] Sutherland C, 2007 A challenge for the development of malaria vaccines: polymorphic target antigens. PLoS Med 4: e116. 17388671 10.1371/journal.pmed.0040116 PMC1820606

[2] Haywood M Conway DJ Weiss H Metzger W D’Alessandro U Snounou G Targett G Greenwood B, 1999 Reduction in the mean number of *Plasmodium falciparum* genotypes in Gambian children immunized with the malaria vaccine SPf66. Trans R Soc Trop Med Hyg 93 (Suppl 1): 65–68. 10.1016/s0035-9203(99)90330-9 10450429

[3] Ouattara A 2013 Molecular basis of allele-specific efficacy of a blood-stage malaria vaccine: vaccine development implications. J Infect Dis 207: 511–519. 23204168 10.1093/infdis/jis709 PMC3537449

[4] Takala SL Plowe CV, 2009 Genetic diversity and malaria vaccine design, testing and efficacy: preventing and overcoming ‘vaccine resistant malaria’. Parasite Immunol 31: 560–573. 19691559 10.1111/j.1365-3024.2009.01138.x PMC2730200

[5] Takala SL 2009 Extreme polymorphism in a vaccine antigen and risk of clinical malaria: implications for vaccine development. Sci Transl Med 1: 2ra5. 10.1126/scitranslmed.3000257 PMC2822345 20165550

[6] Neafsey DE 2015 Genetic diversity and protective efficacy of the RTS,S/AS01 malaria vaccine. N Engl J Med 373: 2025–2037. 26488565 10.1056/NEJMoa1505819 PMC4762279

[7] Douglas AD 2015 A PfRH5-based vaccine is efficacious against heterologous strain blood-stage *Plasmodium falciparum* infection in *Aotus* monkeys. Cell Host Microbe 17: 130–139. 25590760 10.1016/j.chom.2014.11.017 PMC4297294

[8] Ord RL Caldeira JC Rodriguez M Noe A Chackerian B Peabody DS Gutierrez G Lobo CA, 2014 A malaria vaccine candidate based on an epitope of the *Plasmodium falciparum* RH5 protein. Malar J 13: 326. 25135070 10.1186/1475-2875-13-326 PMC4152569

[9] Campeotto I Goldenzweig A Davey J Barfod L Marshall JM Silk SE Wright KE Draper SJ Higgins MK Fleishman SJ, 2017 One-step design of a stable variant of the malaria invasion protein RH5 for use as a vaccine immunogen. Proc Natl Acad Sci USA 114: 998–1002. 28096331 10.1073/pnas.1616903114 PMC5293100

[10] Hayton K 2008 Erythrocyte binding protein PfRH5 polymorphisms determine species-specific pathways of *Plasmodium falciparum* invasion. Cell Host Microbe 4: 40–51. 18621009 10.1016/j.chom.2008.06.001 PMC2677973

[11] Crosnier C 2011 Basigin is a receptor essential for erythrocyte invasion by *Plasmodium falciparum*. Nature 480: 534–537. 22080952 10.1038/nature10606 PMC3245779

[12] Douglas AD Andrews L Draper SJ Bojang K Milligan P Gilbert SC Imoukhuede EB Hill AV, 2011 Substantially reduced pre-patent parasite multiplication rates are associated with naturally acquired immunity to *Plasmodium falciparum*. J Infect Dis 203: 1337–1340. 21459819 10.1093/infdis/jir033 PMC3398130

[13] Bustamante LY Bartholdson SJ Crosnier C Campos MG Wanaguru M Nguon C Kwiatkowski DP Wright GJ Rayner JC, 2013 A full-length recombinant *Plasmodium falciparum* PfRH5 protein induces inhibitory antibodies that are effective across common PfRH5 genetic variants. Vaccine 31: 373–379. 23146673 10.1016/j.vaccine.2012.10.106 PMC3538003

[14] Manske M 2012 Analysis of *Plasmodium falciparum* diversity in natural infections by deep sequencing. Nature 487: 375–379. 22722859 10.1038/nature11174 PMC3738909

[15] Arevalo-Pinzon G Curtidor H Munoz M Patarroyo MA Bermudez A Patarroyo ME, 2012 A single amino acid change in the *Plasmodium falciparum* RH5 (PfRH5) human RBC binding sequence modifies its structure and determines species-specific binding activity. Vaccine 30: 637–646. 22100885 10.1016/j.vaccine.2011.11.012

[16] Tran TM 2014 Naturally acquired antibodies specific for *Plasmodium falciparum* reticulocyte-binding protein homologue 5 inhibit parasite growth and predict protection from malaria. J Infect Dis 209: 789–798. 24133188 10.1093/infdis/jit553 PMC3923542

[17] Tran TM 2013 An intensive longitudinal cohort study of Malian children and adults reveals no evidence of acquired immunity to *Plasmodium falciparum* infection. Clin Infect Dis 57: 40–47. 23487390 10.1093/cid/cit174 PMC3669526

[18] Hall TA, 1999 *BioEdit: A User-Friendly Biological Sequence Alignment Editor and Analysis Program for Windows 95/98/NT*. Oxford, United Kingdom: Oxford University Press, 95–98.

[19] Nei M Gojobori T, 1986 Simple methods for estimating the numbers of synonymous and nonsynonymous nucleotide substitutions. Mol Biol Evol 3: 418–426. 3444411 10.1093/oxfordjournals.molbev.a040410

[20] Tamura K Dudley J Nei M Kumar S, 2007 MEGA4: Molecular Evolutionary Genetics Analysis (MEGA) software version 4.0. Mol Biol Evol 24: 1596–1599. 17488738 10.1093/molbev/msm092

[21] Rozas J, 2009 DNA sequence polymorphism analysis using DnaSP. Methods Mol Biol 537: 337–350. 19378153 10.1007/978-1-59745-251-9_17

[22] Zhang J Nielsen R Yang Z, 2005 Evaluation of an improved branch-site likelihood method for detecting positive selection at the molecular level. Mol Biol Evol 22: 2472–2479. 16107592 10.1093/molbev/msi237

[23] Pond SL Frost SD Muse SV, 2005 HyPhy: hypothesis testing using phylogenies. Bioinformatics 21: 676–679. 15509596 10.1093/bioinformatics/bti079

[24] Hudson RR, 2007 Estimating the recombination parameter of a finite population model without selection. Genet Res 89: 427–432. 18976531 10.1017/S0016672308009610

[25] Kosakovsky Pond SL Posada D Gravenor MB Woelk CH Frost SD, 2006 GARD: a genetic algorithm for recombination detection. Bioinformatics 22: 3096–3098. 17110367 10.1093/bioinformatics/btl474

[26] Kelly JK, 1997 A test of neutrality based on interlocus associations. Genetics 146: 1197–1206. 9215920 10.1093/genetics/146.3.1197 PMC1208047

[27] Rozas J Gullaud M Blandin G Aguade M, 2001 DNA variation at the rp49 gene region of *Drosophila simulans*: evolutionary inferences from an unusual haplotype structure. Genetics 158: 1147–1155. 11454763 10.1093/genetics/158.3.1147 PMC1461709

[28] Huelsenbeck JP Jain S Frost SW Pond SL, 2006 A Dirichlet process model for detecting positive selection in protein-coding DNA sequences. Proc Natl Acad Sci USA 103: 6263–6268. 16606848 10.1073/pnas.0508279103 PMC1458866

[29] Falush D Stephens M Pritchard JK, 2003 Inference of population structure using multilocus genotype data: linked loci and correlated allele frequencies. Genetics 164: 1567–1587. 12930761 10.1093/genetics/164.4.1567 PMC1462648

[30] Lyke KE 2011 Association of HLA alleles with *Plasmodium falciparum* severity in Malian children. Tissue Antigens 77: 562–571. 21447146 10.1111/j.1399-0039.2011.01661.x PMC3152196

[31] Cao K 2004 Differentiation between African populations is evidenced by the diversity of alleles and haplotypes of HLA class I loci. Tissue Antigens 63: 293–325. 15009803 10.1111/j.0001-2815.2004.00192.x

[32] Williams AR 2012 Enhancing blockade of *Plasmodium falciparum* erythrocyte invasion: assessing combinations of antibodies against PfRH5 and other merozoite antigens. PLoS Pathog 8: e1002991. 23144611 10.1371/journal.ppat.1002991 PMC3493472

[33] Chiu CY 2014 Association of antibodies to *Plasmodium falciparum* reticulocyte binding protein homolog 5 with protection from clinical malaria. Front Microbiol 5: 314. 25071730 10.3389/fmicb.2014.00314 PMC4074990

[34] Thera MA 2011 A field trial to assess a blood-stage malaria vaccine. N Engl J Med 365: 1004–1013. 21916638 10.1056/NEJMoa1008115 PMC3242358

[35] Hodder AN Crewther PE Anders RF, 2001 Specificity of the protective antibody response to apical membrane antigen 1. Infect Immun 69: 3286–3294. 11292751 10.1128/IAI.69.5.3286-3294.2001 PMC98287

[36] Genton B 2002 A recombinant blood-stage malaria vaccine reduces *Plasmodium falciparum* density and exerts selective pressure on parasite populations in a phase 1-2b trial in Papua New Guinea. J Infect Dis 185: 820–827. 11920300 10.1086/339342

[37] Obaro SK Adegbola RA Banya WA Greenwood BM, 1996 Carriage of pneumococci after pneumococcal vaccination. Lancet 348: 271–272. 10.1016/s0140-6736(05)65585-7 8684225

[38] Schouls LM van der Ende A van de Pol I Schot C Spanjaard L Vauterin P Wilderbeek D Witteveen S, 2005 Increase in genetic diversity of *Haemophilus influenzae* serotype b (Hib) strains after introduction of Hib vaccination in The Netherlands. J Clin Microbiol 43: 2741–2749. 15956392 10.1128/JCM.43.6.2741-2749.2005 PMC1151946

[39] Baum J Chen L Healer J Lopaticki S Boyle M Triglia T Ehlgen F Ralph SA Beeson JG Cowman AF, 2009 Reticulocyte-binding protein homologue 5–an essential adhesin involved in invasion of human erythrocytes by *Plasmodium falciparum*. Int J Parasitol 39: 371–380. 19000690 10.1016/j.ijpara.2008.10.006

[40] Ouattara A 2010 Lack of allele-specific efficacy of a bivalent AMA1 malaria vaccine. Malar J 9: 175. 20565971 10.1186/1475-2875-9-175 PMC2908102

[41] Sundararaman SA 2016 Genomes of cryptic chimpanzee *Plasmodium* species reveal key evolutionary events leading to human malaria. Nat Commun 7: 11078. 27002652 10.1038/ncomms11078 PMC4804174

[42] Wright KE 2014 Structure of malaria invasion protein RH5 with erythrocyte basigin and blocking antibodies. Nature 515: 427–430. 25132548 10.1038/nature13715 PMC4240730

[43] Barry AE Schultz L Senn N Nale J Kiniboro B Siba PM Mueller I Reeder JC, 2013 High levels of genetic diversity of *Plasmodium falciparum* populations in Papua New Guinea despite variable infection prevalence. Am J Trop Med Hyg 88: 718–725. 23400571 10.4269/ajtmh.12-0056 PMC3617858

[44] Osier FH 2010 Allelic diversity and naturally acquired allele-specific antibody responses to *Plasmodium falciparum* apical membrane antigen 1 in Kenya. Infect Immun 78: 4625–4633. 20732997 10.1128/IAI.00576-10 PMC2976345

[45] Ntege EH Arisue N Ito D Hasegawa T Palacpac NMQ Egwang TG Horii T Takashima E Tsuboi T, 2016 Identification of *Plasmodium falciparum* reticulocyte binding protein homologue 5-interacting protein, PfRipr, as a highly conserved blood-stage malaria vaccine candidate. Vaccine 34: 5612–5622. 27692771 10.1016/j.vaccine.2016.09.028

[46] Galaway F Drought LG Fala M Cross N Kemp AC Rayner JC Wright GJ, 2017 P113 is a merozoite surface protein that binds the N terminus of *Plasmodium falciparum* RH5. Nat Commun 8: 14333. 28186186 10.1038/ncomms14333 PMC5309799

[47] Douglas AD 2011 The blood-stage malaria antigen PfRH5 is susceptible to vaccine-inducible cross-strain neutralizing antibody. Nat Commun 2: 601. 22186897 10.1038/ncomms1615 PMC3504505

[48] Heppner DG Jr. 2005 Towards an RTS,S-based, multi-stage, multi-antigen vaccine against falciparum malaria: progress at the Walter Reed Army Institute of Research. Vaccine 23: 2243–2250. 15755604 10.1016/j.vaccine.2005.01.142

[49] Fluck C Schopflin S Smith T Genton B Alpers MP Beck HP Felger I, 2007 Effect of the malaria vaccine combination B on merozoite surface antigen 2 diversity. Infect Genet Evol 7: 44–51. 16647307 10.1016/j.meegid.2006.03.006

[50] Takala SL 2007 Dynamics of polymorphism in a malaria vaccine antigen at a vaccine-testing site in Mali. PLoS Med 4: e93. 17355170 10.1371/journal.pmed.0040093 PMC1820605

[51] Gandhi K Thera MA Coulibaly D Traore K Guindo AB Ouattara A Takala-Harrison S Berry AA Doumbo OK Plowe CV, 2014 Variation in the circumsporozoite protein of *Plasmodium falciparum*: vaccine development implications. PLoS One 9: e101783. 24992338 10.1371/journal.pone.0101783 PMC4081809

